# Telomerase activity and p53-dependent apoptosis in ovarian cancer cells

**DOI:** 10.1054/bjoc.2001.1812

**Published:** 2001-06

**Authors:** R Akeshima, J Kigawa, M Takahashi, T Oishi, Y Kanamori, H Itamochi, M Shimada, S Kamazawa, S Sato, N Terakawa

**Affiliations:** Department of Obstetrics and Gynecology, Tottori University School of Medicine, 36–1 Nishimachi, Yonago 6838504, Japan

**Keywords:** telomerase, p53, apoptosis, ovarian cancer cells

## Abstract

We conducted the present study to determine the relationship between p53-dependent apoptosis and telomerase activity in ovarian cancer cells. A human ovarian adenocarcinoma cell line, SK-OV-3 that had homozygous deletion of the p53 gene was used in this study. Wild-type p53 genes were transducted to SK-OV-3 cells with a recombinant adenovirus that contained a wild-type p53 gene (AxCAp53). IC _50_ to cisplatin (CDDP) was 12.9 μM for SK-OV-3 cells and 9.2 μM for p53 gene-transducted SK-OV-3 cells. The apoptotic index for cells with p53 gene transduction was significantly higher than cells without transduction. Additionally, p53 gene transduction significantly enhanced CDDP-induced apoptosis. Bax protein in SK-OV-3 cells did not differ before and after exposure to CDDP. In SK-OV-3 cells with transduction of the p53 gene, the expression of p53 and Bax proteins increased after exposure to CDDP. Expression of Bcl-xL decreased after exposure to CDDP in SK-OV-3 cells with and without transduction. The telomerase activity in SK-OV-3 cells with the p53 gene was significantly lower compared with the cells without the p53 gene. CDDP exposure did not affect telomerase activity and human telomerase reverse transcriptase (hTERT) expression in both cell lines. We suggest that the p53 gene may relate to telomerase activity, but that p53-dependent apoptosis does not affect the activity. © 2001 Cancer Research Campaign http://www.bjcancer.com


*British Journal of Cancer* (2001) **84**(11), 1551–1555
© 2001 Cancer Research Campaign
doi: 10.1054/ bjoc.2001.1812, available online at http://www.idealibrary.com on IDEAL®

### Telomerase activity and p53-dependent apoptosis in ovarian cancer cells

**R Akeshima, J Kigawa, M Takahashi, T Oishi, Y Kanamori, H Itamochi, M Shimada, S Kamazawa, S Sato and N Terakawa**

Department of Obstetrics and Gynecology, Tottori University School of Medicine, 36–1 Nishimachi, Yonago 6838504, Japan

**Summary** We conducted the present study to determine the relationship between p53-dependent apoptosis and telomerase activity in ovarian cancer cells. A human ovarian adenocarcinoma cell line, SK-OV-3 that had homozygous deletion of the p53 gene was used in this study. Wild-type p53 genes were transducted to SK-OV-3 cells with a recombinant adenovirus that contained a wild-type p53 gene (AxCAp53). $IC_{50}$ to cisplatin (CDDP) was 12.9 μM for SK-OV-3 cells and 9.2 μM for p53 gene-transducted SK-OV-3 cells. The apoptotic index for cells with p53 gene transduction was significantly higher than cells without transduction. Additionally, p53 gene transduction significantly enhanced CDDP-induced apoptosis. Bax protein in SK-OV-3 cells did not differ before and after exposure to CDDP. In SK-OV-3 cells with transduction of the p53 gene, the expression of p53 and Bax proteins increased after exposure to CDDP. Expression of Bcl-xL decreased after exposure to CDDP in SK-OV-3 cells with and without transduction. The telomerase activity in SK-OV-3 cells with the p53 gene was significantly lower compared with the cells without the p53 gene. CDDP exposure did not affect telomerase activity and human telomerase reverse transcriptase (hTERT) expression in both cell lines. We suggest that the p53 gene may relate to telomerase activity, but that p53-dependent apoptosis does not affect the activity. © 2001 Cancer Research Campaign http://www.bjcancer.com

**Keywords**: telomerase; p53; apoptosis; ovarian cancer cells

Telomeres, which are specialized structures consisting of a large number of tandem repeats TTAGGG at the end of eukaryotic chromosomes, are lost with each cell division. This progressive shortening of telomeres causes chromosomal instability and cell death (Blackburn, 1992). Telomerase, a ribonucleoprotein complex, adds telomeric repeats to the 3′ end of telomeric DNA. This telomere stabilization by telomerase can lead to unlimited cell proliferation (Harley et al, 1995). Human telomerase reverse transcriptase (hTERT), which has been purified as human telomerase catalytic subunits, regulates telomerase activity (Nakayama et al, 1998; Hisatomi et al, 1999).

Abundant evidence indicates that the regulation of telomerase is multifactorial in mammalian cells and involves telomerase gene expression, post-translational protein-protein interactions, and protein phosphorylation. Several proto-oncogenes and tumour suppressor genes have been implicated, both directly and indirectly, in the regulation of telomerase activity (Liu, 1999).

Recent studies showed that normal human mammary epithelial cells transducted with a mutant p53 gene became immortalized and reactive for telomerase, and that wild-type p53 gene transduction inhibited telomerase activity in cancer cells (Chin et al, 1999; Kondo et al, 1998). The p53 gene, which encodes a cell-cycle checkpoint protein, has a pivotal role in inducing apoptosis (Kastan et al, 1995; Fritsche et al, 1993). Those findings suggest a relationship between p53-dependent apoptosis and telomerase activity. However, whether and how p53-dependent apoptosis affect telomerase activity in ovarian cancer is still unknown.

*Received 25 July 2000*
*Revised 15 January 2001*
*Accepted 6 March 2001*

*Correspondence to:* J Kigawa

We conducted the present study to determine the relationship between p53-dependent apoptosis and telomerase activity in ovarian cancer cells.

### MATERIALS AND METHODS

### Cell line

Human ovarian adenocarcinoma cell lines, SK-OV-3 with homozygous deletion of the p53 gene and KF with wild-type p53 gene were used in this study. SK-OV-3 was obtained from the American Type Culture Collection. KF was kindly provided by Dr Kikuchi, National Defense Medical College. SK-OV-3 cells were maintained in minimum essential medium (Nissui, Tokyo, Japan) with 10% fetal bovine serum (FBS) in a humidified atmosphere containing 5% $CO_2$ at 37˚C. KF was maintained in RPMI 1640 medium (Nissui, Tokyo, Japan) with 10% FBS in a humidified atmosphere containing 5% $CO_2$ at 37˚C.

### Cell suppression effect and apoptosis

Wild-type p53 genes were transfected to SK-OV-3 cells with a recombinant adenovirus with wild-type p53 gene (AxCAp53). Infectious units of each virus per cell were 25 MOI for SK-OV-3 according to previous data (Kanamori et al, 1998). A recombinant adenovirus AxCALacZ, encoding for the bacterial LacZ gene under the control of the CAG promoter, was used as a control.

The effect of cisplatin (CDDP) was evaluated by MTT assay in SK-OV-3 cells with and without the p53 gene. Briefly, cells ($10^4$ cells/well) were seeded in 96-well plates and preincubated for 4 h, then incubated for 72 h after exposure to CDDP. Additionally,

SK-OV-3 cells were infected with either AxCAp53 or AxCALacZ. CDDP was added 1 h after infection with the virus, then the cells were incubated for 72 h. Concentrations of CDDP ranged from 0.9 to 22.2 μM. The dose-response curve was plotted on a semi-log scale as a percentage of the control cell number obtained from the untreated sample. The $IC_{50}$ of SK-OV-3 cells was determined from the dose-response curve as a percentage of the control cell number without drugs. The $IC_{50}$ of KF cells (3.1 μM) was determined by our previous data (Takahashi et al, 2000).

For assessment of apoptosis, cells ($10^5$ cells/well) were seeded in 28 $cm^2$ dishes and preincubated for 4 h and CDDP of $IC_{50}$ was added. Twenty-four, 48, and 72 h after CDDP exposure, apoptotic cells were assessed morphologically by staining with Hoechst 33258 (Calbiochem-Novabiochem, San Diego, CA) using cells fixed with Clarke fixative (ethanol: acetic acid = 3:1). The apoptotic index was defined as follows: apoptotic index (%) = 100 × apoptotic cells/1000 cells.

For flow cytometric analysis, cells ($2 \times 10^6$) were collected 24 h and 48 h after treatment. The samples were trypsinized, fixed in 70% ethanol at 4˚C for 1 h, and then resuspended in PBS containing 40 μg/ml propidium iodide and 0.1 mg/ml RNase. After 30 min at 37˚C, the cells were analysed with a FACS Caliber cytofluorometer (Becton Dickinson, San Jose, CA).

### p53, Bax, Bcl-2, and Bcl-xL protein p53, Bax, Bcl-2, and Bcl-xL protein expression were determined by Western blot analysis. $IC_{50}$ to CDDP was exposed 48 h after 25 MOI AxCAp53 infection in SK-OV-3 cells. Twenty-four hours after CDDP exposure, the cells were solubilized on ice in a lysis buffer (50 mM Tris-HCl, 125 mM NaCl, 0.1% NP40, 5 mM ethylenediamine tetraacetic acid, 50 mM NaF, 0.1% phenylmethyl sulfonyl fluoride, and protease inhibitor) and centrifuged at 25 000 × *g* for 30 min. The total protein concentration in the supernatant was measured, and samples of 60 μg protein were separated by electrophoresis on a 4–20% gradient polyacrylamide gel. The separated proteins were transferred onto a polyvinylnylidene difluoride membrane (Milipore Co., Bedford, MA). Those proteins were visualized with antimouse or antirabbit IgG coupled to horseradish peroxidase, using enhanced chemiluminescence according to the manufacturer's recommendation. The primary anti-p53 and Bcl-2 monoclonal antibody were DO-7 (DAKO, Glostrup, Denmark) and Ab-3 (Oncogene Research Product, MA). The anti-Bcl-x and Bax polyclonal antibody were L-19 and N-20 (Santa Cruz Biotechnology, Santa Cruz, CA), respectively. As a control, KF cells were used.

### Telomerase assay

$IC_{50}$ to CDDP was exposed 48 h after 25 MOI AxCAp53 infection in SK-OV-3 cells. Twenty-four, 48, and 72 h after CDDP exposure, samples of SK-OV-3 cells with and without p53 transduction and KF cells were homogenized in 200 μl of 3-[(3-cholamidopropyl) dimethylammonio] propanesulfonic acid (CHAPS) lysis buffer, containing 10 mM Tris-HCl (pH7.5), 1 mM MgCl2, 1 mM EGTA, 0.1 M phenylmethylsulfonyl fluoride, 5 mM 2-mercaptethanol, 0.5% CHAPS, 10% Glycerol, at 4˚C. The suspension was incubated for 30 min on ice and then centrifuged at 15 000 × *g* for 20 min at 4˚C. The protein concentration of the supernatant was determined by Bradford assay (Bradford, 1976).

The telomerase assay was performed using a TRAPEZE Telomerase Detection Kit (Oncor, Gaithersburg, MD, USA). Briefly, cell extracts were assayed in a 50 μl reaction mixture containing 10 × TRAP (telomeric repeat amplification protocol) buffer (0.2 mM Tris-HCl (pH 7.3), 15 mM $MgCl_2$, 630 mM KCl, 0.5% Tween 20, 10 mM EGTA, 0.1% bovine serum albumin), 2.5 mM of each deoxynucleoside triphosphate, 0.1 μg of telomerase substrate primer, 0.1 μg of Primer Mix, 2 units of Taq DNA polymerase and 0.5 μl of CHAPS cell extract. After 10 min of incubation at 30˚C, polymerase chain reaction (PCR) amplification was performed with 30 cycles of 94˚C for 30 s and 60˚C for 30 s. The PCR products were analysed by electrophoresis on 12% polyacrylamide non-denaturating gels and stained with SYBR Green I (Molecular Probes, Eugene, ORE, USA). The gels were photographed using a ATTO Densitograph Lumino-CCD (Atto Corporation, Tokyo, Japan).

A 150-bp DNA standard was used as an internal telomerase assay standard (ITAS). SiHa cells, originated from cervical squamous cell carcinoma, were used as a positive control. Telomerase activity was quantitated on a Macintosh Quadra 840AV computer, using the public domain National Institutes of Health image program (written by Wayne Rasband at the US National Institutes of Health and available from the Internet by anonymous ftp from zippy.nimh.nih.gov or on floppy disk from NTIS, 5285 Port Royal Rd, Springfield, VA 22161 USA, part number PB93-504648). The intensity of telomerase activity was expressed relative to the above-mentioned internal standard.

We also examined hTERT with real time PCR according to Hisatomi et al (1999). Briefly, total RNA was extracted from cells by the acid guanidinium thiocyanate-phenol-chloroform extraction method using Isogen (Wako Junyaku, Osaka, Japan) and DNase I (Takara, Shiga, Japan), and was collected from the precipitate in ethanol. To prepare standard RNA, PCR product was cloned into pBluescript vector (Stratagene Co., La Jolla CA) and was linearised to prevent any activity at the T3 promoter site. Standard RNA was synthesized by using T7 RNA polymerase and was purified by Isogen and DNase I treatment. The PCR reaction mixture was prepared using a TaqMan PCR core reagent kit (PE Applied Biosystems, Norwalk, Conn) according to the manufacturer's instructions. The reaction mixture (50 μl) was prepared containing a final 1 × PCR buffer, 200 mM dATP, 200 mM dCTP, 200 mM dGTP, 500 mM dUTP, 0.5 U AmpErase UNG, 2.5 U AmpliTaq Gold, 5 mM $MgCl_2$, 0.25 μM BABO-F: 5′ -TTTCTACCGGAAGAGTGTCTG-3′ and 0.1 μM BABO-P probe: 5′-CAAGTTGCAAAGCATTG-GAATCAGACA-3′ (GenBank accession number: AF015950). A real-time PCR system (ABI PRISM 7700 Sequence Detection System: PE Applied Biosystems) provided essential information to quantify the initial target copy number according to Heid et al (1996). Using 5′ nuclease activity, a specific fluorescent signal was generated and measured at each cycle during a run. cDNA synthesized with random primer(Gibco BRL, Rockville, MD), and the PCR was made at 50 cycles (94˚C for 30 s, 60˚C for 30 s, 72˚C for 20 s) using primers BABO-F as sense primer and BABO-R as reverse primer. Probes of BABO-P were designed to target an internal region between the primers. The probe was labeled at both ends with fluorescent dyes: fluorescein as a reporter dye and rhodamine as a quencher dye. After annealing the probe onto the internal locus of the amplicon, the probe was cleaved with the 5′ exonuclease activity of thermostable DNA polymerase. After cleaving the probe, the reporter dye emission no longer transferred efficiently to the quencher dye, resulting in an increase in the reporter dye fluorescent emission spectra. The fluorogenic samples were excited with a laser (488 nm) and

 *© 2001 Cancer Research Campaign*

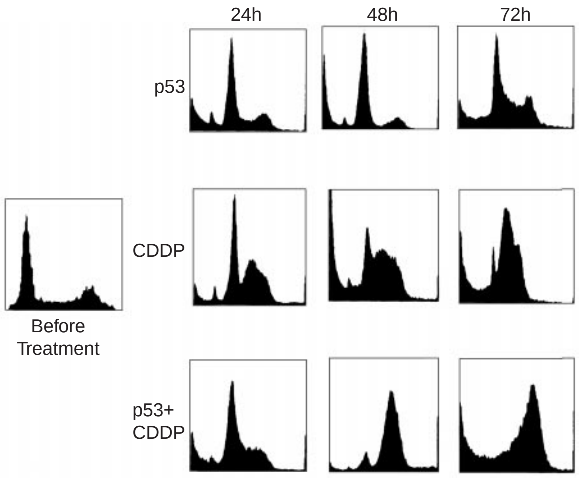

**Figure 1** Flow cytometric analysis. p53 gene transduction induced G1 arrest and cells in pre-G1 phase (apoptosis cells). CDDP increased cells in G2M phase

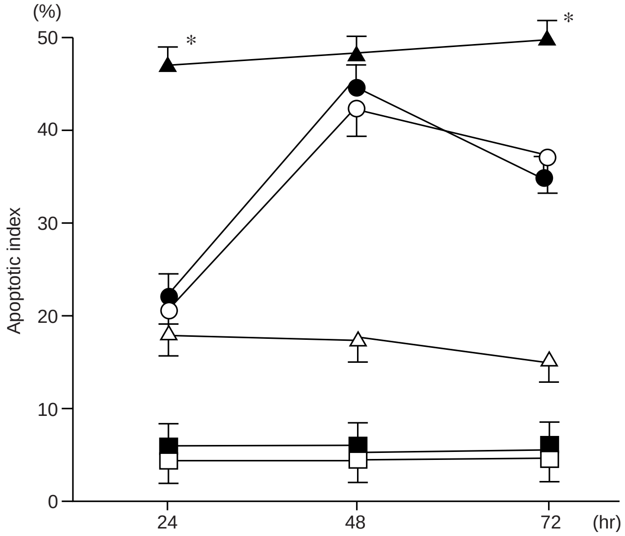

**Figure 2** The apoptotic index were assessed morphologically by staining with Hoechst 33258. CDDP significantly increased the apoptotic index in both SK-OV-3 cells with and without p53 gene transduction. p53 gene transduction significantly enhanced CDDP-induced apoptosis 24 and 72 h after treatment.
▲; CDDP+ p53 ●; CDDP ○; CDDP+AxCALacZ
△; p53 ■; LacZ □; No treatment *; $P < 0.05$

were observed by a charge-coupled device camera during the PCR amplification using an ABI PRISM 7700 Sequence Detection system.

### Statistical analysis

All assays were performed in triplicate. Means for all data were compared by the Student's *t*-test. $P < 0.05$ was considered statistically significant.

### RESULTS

$IC_{50}$ to CDDP was 12.9 μM for SK-OV-3 cells, 11.8 μM for AxCALacZ infected SK-OV-3 cells, and 9.2 μM for p53 gene transducted SK-OV-3 cells. In flow cytometric analysis, p53 gene transduction induced G1 arrest and cells in pre-G1 phase (apoptosis cells). CDDP increased cells in G2M phase. p53 gene transduction enhanced the effect of CDDP (Figure 1). Apoptotic index for cells with p53 gene transduction was significantly higher, compared with cells without transduction. CDDP significantly increased the apoptotic index in both SK-OV-3 cells with and without p53 gene transduction. The apoptotic index increased and reach to the peak 48 h after CDDP exposure. p53 gene transduction significantly enhanced CDDP-induced apoptosis 24 and 72 h after treatment (Figure 2).

Figure 3 shows the expression of p53 and p53-associated proteins in each cell line. p53 protein was expressed after AxCAp53 infection and enhanced by exposure to CDDP. Bax protein in SK-OV-3 cells did not differ before and after exposure to CDDP. In SK-OV-3 cells with transduction of the p53 gene, Bax protein expression increased after exposure to CDDP. Expression of Bcl-xL decreased after exposure to CDDP in SK-OV-3 cells with and without transduction. In KF cells, p53 and Bax protein expression increased, but expression of Bcl-xL slightly decreased after exposure to CDDP. The expression of Bcl-2 protein did not change after exposure to CDDP in either cell line.

The telomerase activity did not differ among 24, 48, and 72 h after each treatment. The intensity of telomerase activity was 1.05 ± 0.10 for no exposure control, 1.02 ± 0.13 for CDDP exposure in SK-OV-3

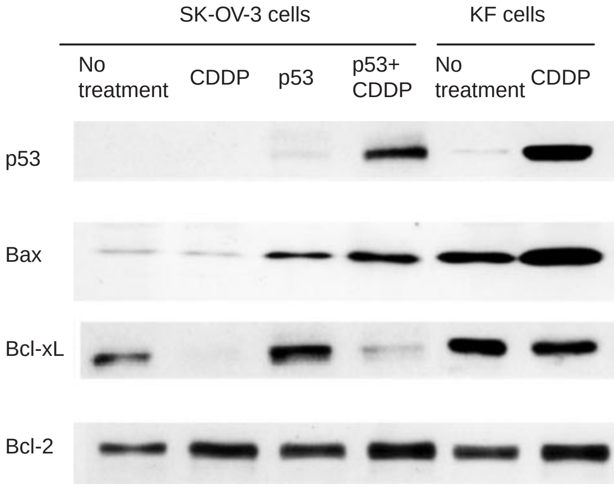

**Figure 3** The expression of p53 and p53-associated proteins in each cell line. p53 protein expressed after AxCAp53 infection and enhanced by exposure to CDDP. Bax protein in SK-OV-3 cells did not differ before and after exposure to CDDP. In SK-OV-3 cells with transduction of p53 gene and KF cells, Bax protein expression increased after exposure to CDDP. Expression of Bcl-xL decreased after exposure to CDDP in SK-OV-3 cells with and without transduction cells, and 0.76 ± 0.10 and 0.78 ± 0.14, respectively, in SK-OV-3 cells with transduction of p53 gene (Figure 3, 4 A). The hTERT expression also did not change with time after treatment. The expression of hTERT mRNA (log copies / μg total RNA) was 4.4 for no exposure control, 4.7 for CDDP exposure in SK-OV-3 cells, and 3.8 and 3.9, respectively, in SK-OV-3 cells with transduction of the p53 gene (Figure 4 B). The telomerase activity in SK-OV-3 cells with the p53 gene was significantly lower compared with the cells without the p53 gene. The telomerase activity for AxCALacZ did not differ from that

© 2001 Cancer Research Campaign

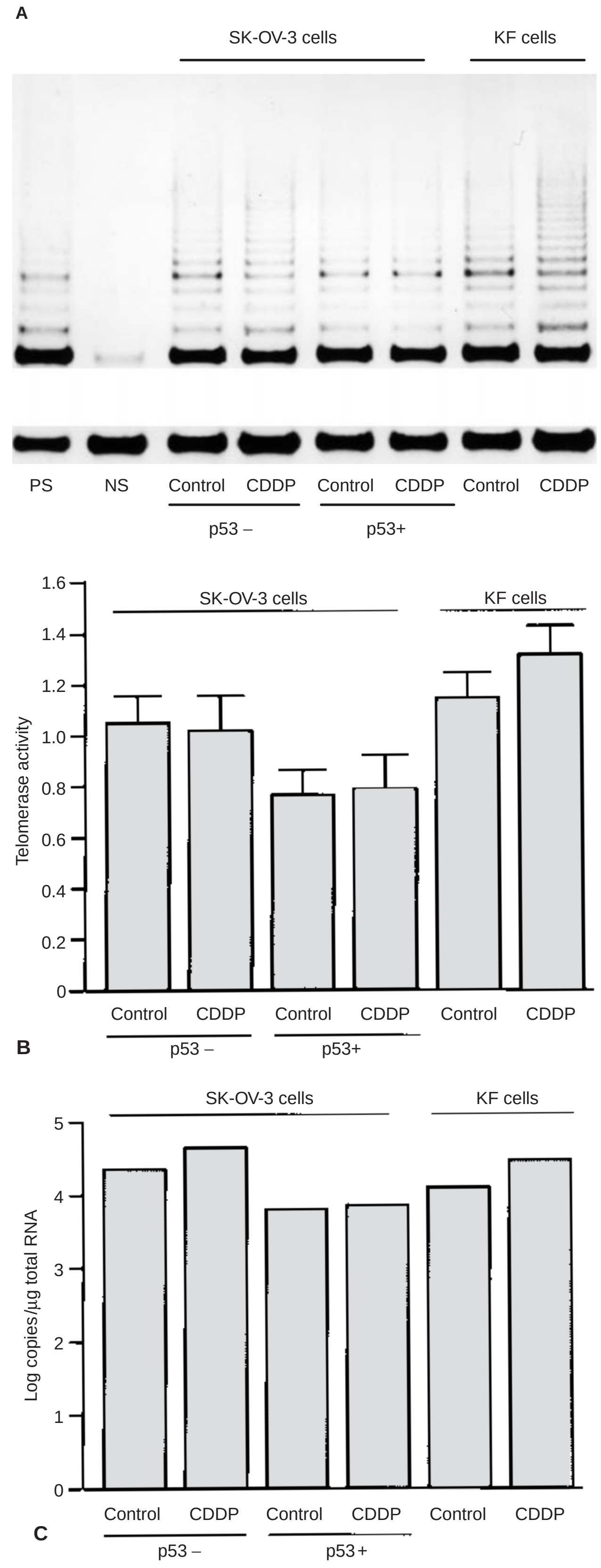

**Figure 4** Telomerase activity measured by TRAP assay (**A**) 24 h after exposure to CDDP. The intensity was quantitated by NIH image (**B**). Telomerase activity in SK-OV-3 cells with p53 gene was significantly lower compared with the cells without the p53 gene. CDDP exposure did not affect telomerase activity or hTERT expression in either cell line (**C**)

of non-infected SK-OV-3 cells. CDDP exposure did not affect telomerase activity and hTERT expression in either cell line.

### DISCUSSION

Although several authors have examined the relationship between p53 and telomerase activity, the results have been contradictory (Kusumoto et al, 1999; Maxwell et al, 1997; Righetti et al, 1996). Kusumoto et al demonstrated that the p53 gene transduction directly inhibited telomerase activity in pancreatic cancer cells (1999). In contrast, another author showed that telomerase activity was found to be unaffected by overexpression of p53 in immortalized endothelial cells (1997). Those studies investigated telomerase activity in cells that had overexpression of p53. For the present study, we compared telomerase activity after transduction of the p53 gene and p53-deficient cells to determine whether p53-dependent apoptosis affects telomerase activity in ovarian cancer. In our previous study, the efficiency of the recombinant adenovirus to transduce SK-OV-3 cells was 89% for 6.25 MOI, 94% for 12.5 MOI and 100% for over 25 MOI (Kanamori et al, 1998). Based on the data, we chose 25 MOI of AxCAp53 to avoid overexpression of p53. Additionally, a human ovarian adenocarcinoma cell lines, KF cells which had wild-type p53 gene were used as a control.

In the present study, telomerase activity decreased after transduction of the p53 gene. This result supports the likelihood that the p53 gene contributed to the shortening of telomeres in a p53-negative H1299 human non-small cell lung cancer cell line (Mukhopadhyay et al, 1998). p53 gene transduction not only induced apoptosis but also caused G1 arrest. Because the p21 gene that arrested the cell cycle at the G1 phase did not affect telomerase activity (Kusumoto et al, 1999), p53-dependent apoptosis may directly relate to telomerase activity.

SK-OV-3 cells with p53 gene transduction had higher sensitivity to CDDP than did those without the p53 gene. Additionally, p53 gene transduction enhanced CDDP-induced apoptosis, supporting the previous findings that the p53 gene contributes to sensitivity to CDDP (Kastan et al, 1995; Kanamori et al, 1998; Sato et al, 1999). Regardless of p53 gene status, the apoptotic index increased after exposure to CDDP, and apoptosis paralleled cytotoxity in both cell lines. After exposure to CDDP, Bax protein, which is directly regulated by p53 gene, increased in KF and p53 gene-transducted SK-OV-3 cells, but not in SK-OV-3 cells without the p53 gene. The Bcl-2 family competes for Bax homodimerization and forms heterodimers. Bcl-xL, which is one of the Bcl-2 family, has an important role in solid tumours, (Marone et al, 1998). Bcl-xL, a functional and structural homologue of Bcl-2, provides protection from apoptosis (Boise et al, 1993; Henriksen et al, 1995; Marone et al, 1998). In the present study, the expression of Bcl-xL decreased after exposure to CDDP in all cell lines. KF cells have a p53-dependent pathway and SK-OV-3 cells, which had homozygous deletion of the p53 gene, may have a p53-independent pathway. In p53 gene-transducted SK-OV-3 cells, CDDP-induced apoptosis occurred through p53-dependent and -independent pathways. The appearance of p53-dependent apoptotic pathway may cause the higher sensitivity to CDDP in p53-transducted cells. Although it is reported that stable over-expression of Bcl-2 in cancer cells is accompanied by increased levels of telomerase activity (Mandal et al, 1997), Bcl-2 expression did not differ among all cell lines.

 *© 2001 Cancer Research Campaign*

CDDP causes DNA strand breaks especially at guanine residues followed by apoptosis (Pil et al, 1997). TRAP assay measures telomerase activity within an artificial system and does not measure the in vitro activity. It is unlikely that CDDP would have no effect on telomerase in TRAP assay. Therefore, we also examined hTERT with real time PCR. A recent study showed that CDDP reduced telomerase activity in human testicular tumor cells (Burger et al, 1997). In our series, CDDP did not affect telomerase activity or hTERT expression in all cell lines. Another study showed that telomerase activity decreased 24–48 h after exposure to CDDP, but the phenomenon might relate to cell viability (Akiyama et al, 1999). In contrast our data demonstrated that both telomerase activity and hTERT expression did not change with time after treatment. Consequently, CDDP-induced apoptosis via p53-dependent and -independent pathway apoptosis did not affect telomerase activity in ovarian cancer cells.

Deletion of p53 significantly attenuated the adverse cellular effects of telomere dysfunction (Chin et al, 1999). In the present study, the telomere length of SK-OV-3 cells did not differ between cells with and those without p53 gene transduction (data not shown), although SK-OV-3 cells showed higher telomerase activity compared with cells with p53 gene transduction. The duration may have been too short to change telomere length in our series.

In conclusion, the p53 gene may relate to telomerase activity, but p53-gene-dependent apoptosis does not affect this activity.

### ACKNOWLEDGEMENTS

We thank Mr Mitsuo Oshimura PhD, Mr Motonobu Kato and Mr Arata Nishimoto (Department of Molecular and Cell Genetics, School of Life Science, Faculty of Medicine, Tottori University) for kindly providing ITAS, and helpful technical advice.

### REFERENCES

Akiyama M, Horiguchi YJ, Saito S, Yamada O, Mizoguchi H and Yamada H (1999) Cytostatic concentration of anticancer agents do not affect telomerase activity of leukaemic cells in vitro. *Eur J Cancer* **35**: 309–315

Blackburn EH (1992) Telomerases. *Annu Rev Biochem* **61**: 113–119

Boise LH, Gonzalez-Garcia M, Postema CE, Ding L, Lindsten T, Turka LA, Mao X, Nunez G and Thompson CB (1993) Bcl-x, a bcl-2-related gene that functions as a dominant regulator of apoptotic cell death. *Cell* **74**: 597–608

Bradford MM (1976) A rapid and sensitive method for the quantitation of microgram quantities of protein utilizing the principle of protein-dye binding. *Anal Biochem* **72**: 248–254

Burger AM, Double JA and Newell DR (1997) Inhibition of telomerase activity by cisplatin in human testicular cancer cells. *Eur J Cancer* **33**: 638–644

Chin L, Artandi SE, Shen Q, Tam A, Lee SL, Gottlieb GJ, Greider CW and DePinho RA (1999) p53 deficiency rescues the adverse effects of telomere loss and cooperates with telomere dysfunction to accelerate carcinogenesis. *Cell* **97**: 527–538

Fritsche M, Haessler C and Brandner G (1993) Induction of nuclear accumulation of the tumor-suppressor protein p53 by DNA-damaging agents. *Oncogene* **8**: 307–318

Harley CB, Kim NW, Prowse KR, Weinrich SL, Hirschi K, West MD, Bacchetti S, Hirte HW, Counter CM and Greider CW (1995) Telomerase, cell immortality and cancer. *Cold Spring Harbor Symp Quant Biol* **59**: 307–315

Heid AC, Stevebs J, Livak KJ and Williams PM (1996) Real time quantitative PCR. *Genome Res* **6**: 986–994

Henriksen R, Wilander E and Oberg K (1995) Expression and prognostic significance of Bcl-2 in ovarian tumours. *Br J Cancer* **72**: 1324–1329

Hisatomi H, Nagao K, Kanamaru T, Endo H, Tomimatsu M, Hikij K (1999) Levels of telomerase catalytic subunit mRNA as a predictor of potential malignancy. *Int J Oncol* **14**: 727–732

Kanamori Y, Kigawa J, Minagawa Y, Irie T, Oishi T, Shimada M, Takahashi M, Nakamura T, Sato K and Terakawa N (1998) A newly developed adenovirus-mediated transfer of a wild-type p53 gene increases sensitivity to cis-diamminedichloroplatinum (II) in p53-deleted ovarian cancer cells. *Eur J Cancer* **34**: 1802–1806

Kastan MB, Canman CE and Leonard CJ (1995) p53 cell cycle control and apoptosis: implications for cancer. *Cancer Metastasis Rev* **14**: 3–15

Kondo Y, Kondo S, Tanaka Y, Haqqi T, Barna BP and Cowell JK (1998) Inhibition of telomerase increases the susceptibility of human malignant, glioblastoma cells to cisplatin-induced apoptosis. *Oncogene* **30; 16**: 2243–2248

Kusumoto M, Ogawa T, Mizumoto K, Ueno H, Niiyamai H, Sato N, Nakamura M and Tanaka M (1999) Adenovirus-mediated p53 gene transduction inhibits telomerase activity independent of its effects on cell cycle arrest and apoptosis in human pancreatic cancer cells. *Clin Cancer Res* **5**: 2140–2147

Liu JP (1999) Studies of the molecular mechanisms in the regulation of telomerase activity. *FASEB J* **13**: 2091–2104

Mandal M and Kumar R (1997) Bcl-2 modulates telomerase activity. *J Biol Chem* **272**: 14183–14187

Marone M, Scambia G, Mozzetti S, Ferrandia G, Iacovella S, DePasqua A, Benedetti Panici P and Mancuso S (1998) Bcl-2, bax, bcl-XL, and bcl-XS expression in normal and neoplastic ovarian tissues. *Clin Cancer Res* **4**: 517–524

Maxwell SA, Capp D and Acosta SA (1997) Telomerase activity in immortalized endothelial cells undergoing p53-mediated apoptosis. *Biochem Biophys Res Commun* **241**: 642–645

Mukhopadhyay T, Multani AS, Roth JA and Pathak S (1998) Reduced telomeric signals and increased telomeric associations in human lung cancer cell lines undergoing p53-mediated apoptosis. *Oncogene* **17**: 901–906

Nakayama J, Tahara H, Tahara E, Saito M, Ito K, Nakamura H, Nakanishi T, Tahara E, Ide T and Ishikawa F (1998) Telomerase activation by hTRT in human normal fibroblasts and hepatocellular carcinomas. *Nat Genet* **18**: 65–68

Pil P and Lippard SJ (1997) *Cisplatin and related drugs*, pp 392–410. Academic: San Diego

Righetti SC, Della Torre G, Pilotti S, Menard S, Ottone F, Colnaghi MI, Pierotti MA, Lavarino C, Cornarotti M, Oriana S, Bohm S, Bresciani GL, Spatti G and Zunino F (1996) A comparative study of p53 gene mutations, protein accumulation, and response to cisplatin-based chemotherapy in advanced ovarian carcinoma. *Cancer Res* **56**: 689–693

Sato S, Kigawa J, Minagawa Y, Okada M, Shimada M, Takahashi M, Kamazawa S and Terakawa N (1999) Chemosensitivity and p53-dependent apoptosis in epithelial ovarian carcinoma. *Cancer* **86**: 1307–1313

Takahashi M, Kigawa J, Minagawa Y, Itamochi H, Shimada M, Kamazawa S, Sato S, Akeshima R and Terakawa N (2000) Sensitivity to paclitaxel is not related to p53-dependent apoptosis in ovarian cancer cells. *Eur J Cancer* **36**: 1863–1868

© 2001 Cancer Research Campaign 
